# Development of Simple and Accurate in Silico Ligand-Based Models for Predicting ABCG2 Inhibition

**DOI:** 10.3389/fchem.2022.863146

**Published:** 2022-05-18

**Authors:** Shuheng Huang, Yingjie Gao, Xuelian Zhang, Ji Lu, Jun Wei, Hu Mei, Juan Xing, Xianchao Pan

**Affiliations:** ^1^ Department of Medicinal Chemistry, School of Pharmacy, Southwest Medical University, Luzhou, China; ^2^ Key Laboratory of Biorheological Science and Technology (Ministry of Education), College of Bioengineering, Chongqing University, Chongqing, China; ^3^ Department of Pathophysiology, School of Basic Medical Science, Southwest Medical University, Luzhou, China

**Keywords:** ABCG2 (BCRP), *in silico*, prediction, inhibitors, PLS-DA

## Abstract

The ATP binding cassette transporter ABCG2 is a physiologically important drug transporter that has a central role in determining the ADMET (absorption, distribution, metabolism, elimination, and toxicity) profile of therapeutics, and contributes to multidrug resistance. Thus, development of predictive in silico models for the identification of ABCG2 inhibitors is of great interest in the early stage of drug discovery. In this work, by exploiting a large public dataset, a number of ligand-based classification models were developed using partial least squares-discriminant analysis (PLS-DA) with molecular interaction field- and fingerprint-based structural description methods, regarding physicochemical and fragmental properties related to ABCG2 inhibition. An in-house dataset compiled from recently experimental studies was used to rigorously validated the model performance. The key molecular properties and fragments favored to inhibitor binding were discussed in detail, which was further explored by docking simulations. A highly informative chemical property was identified as the principal determinant of ABCG2 inhibition, which was utilized to derive a simple rule that had a strong capability for differentiating inhibitors from non-inhibitors. Furthermore, the incorporation of the rule into the best PLS-DA model significantly improved the classification performance, particularly achieving a high prediction accuracy on the independent in-house set. The integrative model is simple and accurate, which could be applied to the evaluation of drug-transporter interactions in drug development. Also, the dominant molecular features derived from the models may help medicinal chemists in the molecular design of novel inhibitors to circumvent ABCG2-mediated drug resistance.

## 1 Introduction

ABCG2, also known as breast cancer resistance protein (BCRP), is a physiologically important transporter of ATP-binding-cassette (ABC) superfamily. It is constitutively expressed and distributed on the cell surfaces of various tissues and barriers, including the mammary glands, liver, kidney, the blood-brain, blood-testis and maternal-fetal barriers, where it plays a secretory role or a major protective role against xenobiotics (Fetsch et al., 2006; Robey et al., 2009).

At structural level, ABCG2 is comprised of two conserved nucleotide binding domains (NBDs) responsible for ATP binding and hydrolysis, and two transmembrane domains (TMDs) that can form the drug-binding pocket and transport pathway (Taylor et al., 2017). Similar to its functional homologs ABCB1 (P-glycoprotein) and ABCC1 (MRP1), ABCG2 acts as a promiscuous drug efflux pump with broad substrate specificity. Powered by ATP hydrolysis, the pump can transport a wide range of commonly used drugs out of the cell, thus greatly affecting their pharmacokinetic parameters and clinical dispositions (Mao and Unadkat, 2015; Iorio et al., 2016). As a result, this transporter is on the US Food and Drug Administration and the European Medicines Agency lists of transporters to be checked for clinically relevant drug-drug interactions.

Most importantly, many chemotherapeutic agents with diverse structures and chemical properties such as mitoxantrone, camptothecin analogues, epipodophyllotoxin analogues, methotrexate, and tyrosine kinase inhibitors, are known to be ABCG2 substrates (Eckford and Sharom, 2009). Consequently, the overexpression of ABCG2 found in many human cancers is thought to be a major contributor to the development of multidrug resistance (MDR), which is a serious obstacle in cancer treatment (Fletcher et al., 2016; Robey et al., 2018). As an attractive molecular target to overcome MDR, efforts were directed at developing ABCG2 inhibitors that have been rationalized as adjuvant therapy when coadministered with anticancer drugs (Li W. et al., 2016). Despite showing promise in cell models, most of the candidates failed in clinical trials due to poor selectivity, unsatisfactory efficacy, or excessive toxicity (Kathawala et al., 2015). For these reasons, it is of particular importance to predict and evaluate ABCG2 inhibition early in the drug discovery pipeline.

Reliable *in vitro* assays to evaluate ABCG2 inhibition are very costly and time-consuming. By contrast, computational quantitative structure-activity relationship (QSAR) models provide a fast and cost-efficient approach to achieve this goal. The large, featureless, and highly lipophilic binding sites, together with the high flexibility of the structure, justify the prevalence of ligand-based computational models. Recently, many research efforts have been directed to the development of in silico models for the prediction of ABCG2 inhibition. For instance, Pan et al. created Bayesian classification models based on a training set of 124 ABCG2 inhibitors and non-inhibitors and pharmacophore models based on 30 potent ABCG2 inhibitors, with the best models achieving overall prediction accuracies of 90% and 66%, respectively, for a same test set including 79 samples (Pan et al., 2013). In a later study, Montanari and Ecker collected a data set of 978 ABCG2 inhibitors and non-inhibitors from 47 sources, and reported a Bayesian classification model with an accuracy of 91.9% under 10-fold for the 780 training samples (Montanari and Ecker, 2014). Recently, Belekar et al. developed various classification models based on 197 training samples by using machine learning (ML) methods including support vector machine (SVM), k-nearest neighbor (k-NN), and artificial neural networks (ANN), yielding global accuracies in the 82.8–87.8% range for the 99 test samples and 74.5–77.5% for the 99 validation samples (Belekar et al., 2015). More recently, by reusing the dataset of 978 compounds, Montanari et al. built a logistic regression model with a MCC (Matthews correlation coefficient) of 0.65 and an AUC (the area under the receiver operating characteristic curve) of 0.90 on the training set (Montanari et al., 2017). Despite being very valuable for researches, the generalization and application of these models are somewhat limited by the size of the dataset (often less than 1,000 compounds in total) with confined chemical space coverage and complicated modeling procedures. Furthermore, the effectiveness of these models for unknown data remains little known due to the lack of an external validation. Therefore, development of simple, interpretable, and accurate models has always been pursued, aiming to generate easily understandable guidelines that allow to evaluate drug-transporter interactions and design lead inhibitors for clinical trials.

In this work, based on a publicly available dataset of 1,104 compounds, an integrative model was proposed to predict ABCG2 inhibition by using molecular hologram based partial least-squares discriminant analysis (PLS-DA) combined with a simple rule derived from an informative VolSurf descriptor. In particular, an in-house dataset curated from 35 experimental studies was used to further validate the model performance against unknown data. Furthermore, important chemical and structural properties beneficial to ABCG2 inhibition were discussed in detail, which were verified by structure-based molecular docking studies. The information derived from the predictive in silico models could be used to guide the molecular design of ABCG2 inhibitors.

## 2 Materials and Methods

### 2.1 Dataset

A public dataset of 1,104 compounds (533 inhibitors and 571 non-inhibitors) compiled by Montanari et al. was used to develop the classification models for predicting ABCG2 inhibition (Montanari et al., 2016). Briefly, they integrated the previously published dataset (433 inhibitors and 545 non-inhibitors) into the data retrieved from the Open PHACTS Discovery Platform (473 inhibitors and 144 non-inhibitors) by a semi-automatic, fully flexible KNIME workflow. In this work, the dataset was divided into a training set (355 inhibitors and 381 non-inhibitors) and an internal validation set (178 inhibitors and 190 non-inhibitors). To maximize structural diversity and chemical coverage, the 736 training compounds for model establishment were singled out from a small molecule library analysis using the Find Diverse Molecules protocol in Discovery Studio (version 2.5) software. The Tanimoto distances between the iteratively selected samples were evaluated based on ECFP_6 fingerprints for subset selection. The remaining 368 compounds severed as an internal validation set was employed to evaluate the predictive power of the obtained classification models.

Specially, an external validation set was manually curated from 35 recent publications (Gallus et al., 2014; Gu et al., 2014; Shukla et al., 2014; Tan et al., 2014; Winter et al., 2014; Yang et al., 2014; Gozzi et al., 2015; Koehler and Wiese, 2015; Li et al., 2015; Marighetti et al., 2015; Chen et al., 2016; Gupta et al., 2016; Kraege et al., 2016a; Kraege et al., 2016b; Krapf and Wiese, 2016; Li et al., 2016b; Miyata et al., 2016; Pires et al., 2016; Reznicek et al., 2016; Schmitt et al., 2016; Schwarz et al., 2016; Song et al., 2016; Spindler et al., 2016; Gujarati et al., 2017; Krapf et al., 2017b; Krapf et al., 2017a; Marchitti et al., 2017; Montanari et al., 2017; Schaefer et al., 2017; Sjöstedt et al., 2017; Stefan et al., 2017; Zhang et al., 2017; Koehler et al., 2018; Liao et al., 2018; Paterna et al., 2018). Because experimental assays were performed under different conditions (e.g., concentration of compound and cell models), it was impossible to set up an inhibition threshold for the definition of inhibitors or non-inhibitors. Thus, a compound was identified as an inhibitor or a non-inhibitor according to the criterion created by the authors in each original research. The compounds with ambiguous activities or already present in the public dataset were discarded, yielding an in-house dataset of 634 compounds (500 inhibitors and 134 non-inhibitors), which was used to further evaluate the predictive power of the models against unknown data. The activity of all compounds was represented by a binary variable (1 for inhibitor, 0 for non-inhibitor). The datasets of 1738 compounds are listed in Supplementary Table S1.

After removing counterions and adding hydrogens, each molecule was charged by MMFF94 method and then optimized by Tripos force field with conjugate gradient minimizer built-in Sybyl (version 8.1) package. The maximum iteration steps and energy gradient were set to 5,000 times and 0.05 kcal⋅mol^−1^⋅Å^−1^, respectively.

### 2.2 Structural Description

#### 2.2.1 VolSurf Description

VolSurf descriptors represent the physicochemical properties for a given set of molecules by utilizing GRID molecular interaction fields (Cruciani et al., 2000). In Volsurf calculation, each grid vertex around a molecule is detected with chemical probes, and then most of the relevant information present in the 3D molecular fields map are compressed into a few 2D numerical descriptors, which can quantitatively characterize the molecular size and shape, the size and shape of both hydrophilic and hydrophobic regions, and the balance between them. Because of the nature of descriptors, VolSurf is primarily independent of conformational alignment of molecules. Currently, a total of nine probes can be used in VolSurf, including water (OH2), a hydrophobic probe (DRY), an amphipathic probe (BOTH), H-bonding carbonyl (O), sp2 carboxy oxygen atom (O:), sp2 phenolate oxygen (O−), neutral flat NH (N1), sp2 N with one lone pair (N: = ), and sp3 amine NH3 cation (N3+). Among them, the OH2 and DRY probes are generally used in most cases, which define the hydrophilic and hydrophobic regions, respectively. Other probes can be selectively used in certain cases.

In this work, a total of 118 VolSurf descriptors were generated using five chemical probes (OH2, DRY, BOTH, O, and O:) based on the 736 diverse training compounds. To elicit the most discriminative molecular descriptors, feature selection was performed using stepwise linear regression analysis, in which the variables are introduced into the model one by one and evaluated by F-test at each iteration. The initially introduced variables may become no longer significant due to the introduction of later variables, which will be removed to ensure that only significant variables are included in the regression equation. In this case, the entry and removal probability of F value were set to 0.02 and 0.10, respectively.

#### 2.2.2 Molecular Hologram

The molecular hologram is a fragment based molecular description method in which the structural information of a molecule can be transformed into a molecular fingerprint (Hurst and Heritage, 1997). First, molecules are broken into predefined structural fragments. Then, each unique fragment is assigned a specific large integer by means of cyclic redundancy check (CRC) algorithm. Each integer corresponds to a bin in an integer array of fixed length L. Bin occupancies are incremented according to the fragments generated. All generated fragments are hashed into array bins in the range 1 to L. The array containing counts of molecular fragments is molecular hologram, and bin occupancies are the hologram descriptors. Compared to the traditional 2D fingerprints, molecular hologram contains additional information such as branched and cyclic fragments and stereochemistry of the molecule. Molecular hologram description was carried out by Hologram QSAR (HQSAR) module of Sybyl (version 8.1) package.

### 2.3 PLS-DA Modeling and Performance Evaluation

Partial least squares-discriminant analysis (PLS-DA) is a supervised machine learning method with full awareness of the class labels that has been widely used in the field of cheminformatics. It can be used for dimensionality-reduction, feature selection as well as classification task. Therefore, PLS-DA is considered as a supervised version of traditional principal component analysis (PCA). Particularly, PLS-DA is a proper technique to explore pattern recognition or to develop classification models, respectively, when the molecules are described by Volsurf descriptors or molecular holograms. It utilizes a projection space-based statistical method that combines PCA and multiple linear regression, which aims to find a separating hyperplane and divides the space into two regions (Lee et al., 2018). It should be noted that since PLS-DA and other machine learning methods such as support vector machine (SVM) are prone to overfitting, cross-validation is an indispensable step in the construction of a classifier.

In PLS-DA modeling, all variables were auto-scaled and the number of principal components was determined by 10-fold cross-validation. The performance of the established models was evaluated based on the true positives (TP), true negatives (TN), false positives (FP), and false negatives (FN) by the standard performance measures: accuracy (ACC), sensitivity (SEN), specificity (SPE), and the Matthews correlation coefficient (MCC). These measures are calculated according to Eqs 1–4.

$$\mathrm{ACC}=\frac{\mathrm{TP}+\mathrm{TN}}{\mathrm{TP}+\mathrm{FP}+\mathrm{TN}+\mathrm{FN}}\;\;\;\;\;\;0\leq \mathrm{ACC}\leq 1$$ (1)

$$\mathrm{SEN}=\frac{\mathrm{TP}}{\mathrm{TP}+\mathrm{FN}}\;\;\;\;\;0\leq \mathrm{SEN}\leq 1$$ (2)

$$\mathrm{SPE}=\frac{\mathrm{TN}}{\mathrm{TN}+\mathrm{FP}}\;\;\;\;\;0\leq \mathrm{SPE}\leq 1$$ (3)

$$\mathrm{MCC}=\frac{\mathrm{TP}\times \mathrm{TN}-\mathrm{FN}\times \mathrm{FP}}{\sqrt{\left(\mathrm{TN}+\mathrm{FN}\right)\left(\mathrm{FN}+\mathrm{TP}\right)\left(\mathrm{TP}+\mathrm{FP}\right)\left(\mathrm{FP}+\mathrm{TN}\right)}}\;\;\;\;\;-1\leq \mathrm{MCC}\leq 1$$ (4)

where ACC equals the overall accuracy for all compounds; SEN and SPE indicate the model performance in correctly identifying inhibitors and non-inhibitors, respectively; and MCC value ranges from −1 and 1. A higher MCC value means a better prediction for the true positives and negatives.

### 2.4 Molecular Docking

The fully automatic flexible Surflex-dock built-in Sybyl 8.1 was employed for docking studies (Jain, 2003). The conformational search in Surflex-Dock was guided by a ‘protomol’, an ensemble of small probes (CH4, NH and CO) that make favorable interactions with a predefined binding site. The crystal structure of ABCG2 in inhibitor-bound state, with a resolution of 3.56 Å, was retrieved from the Protein Data Bank (PDB ID: 6FFC). Prior to docking, the protein and compounds were optimized by Amber and Tripos force fields, respectively. The residues within the 4 Å distance from the co-crystallized inhibitor MZ-29 were used to generate the protomol by using a “thresh” of 0.5 and a “bloat” of 3. During the docking process, the flexibility of side chains within 4 Å distance from a ligand was allowed to adapt the conformation of the docked ligand. In a post-processing step, the conformation of each ligand was further optimized in the context of the receptor by using a BFGS quasi-Newton method and an internal Dreiding force field. The docking poses of each ligand were sorted by Total Scores expressed in -log10 (Kd) unit, which consists of hydrophobic, polar, electrostatic, repulsive, entropic, solvation and crash terms. For each ligand, both the number of starting conformations and retained docking poses were set to 20. Other docking parameters were set by default.

## 3 Results and Discussion

### 3.1 PLS-DA Modeling

#### 3.1.1 Volsurf-Based Models

**FIGURE 1:**
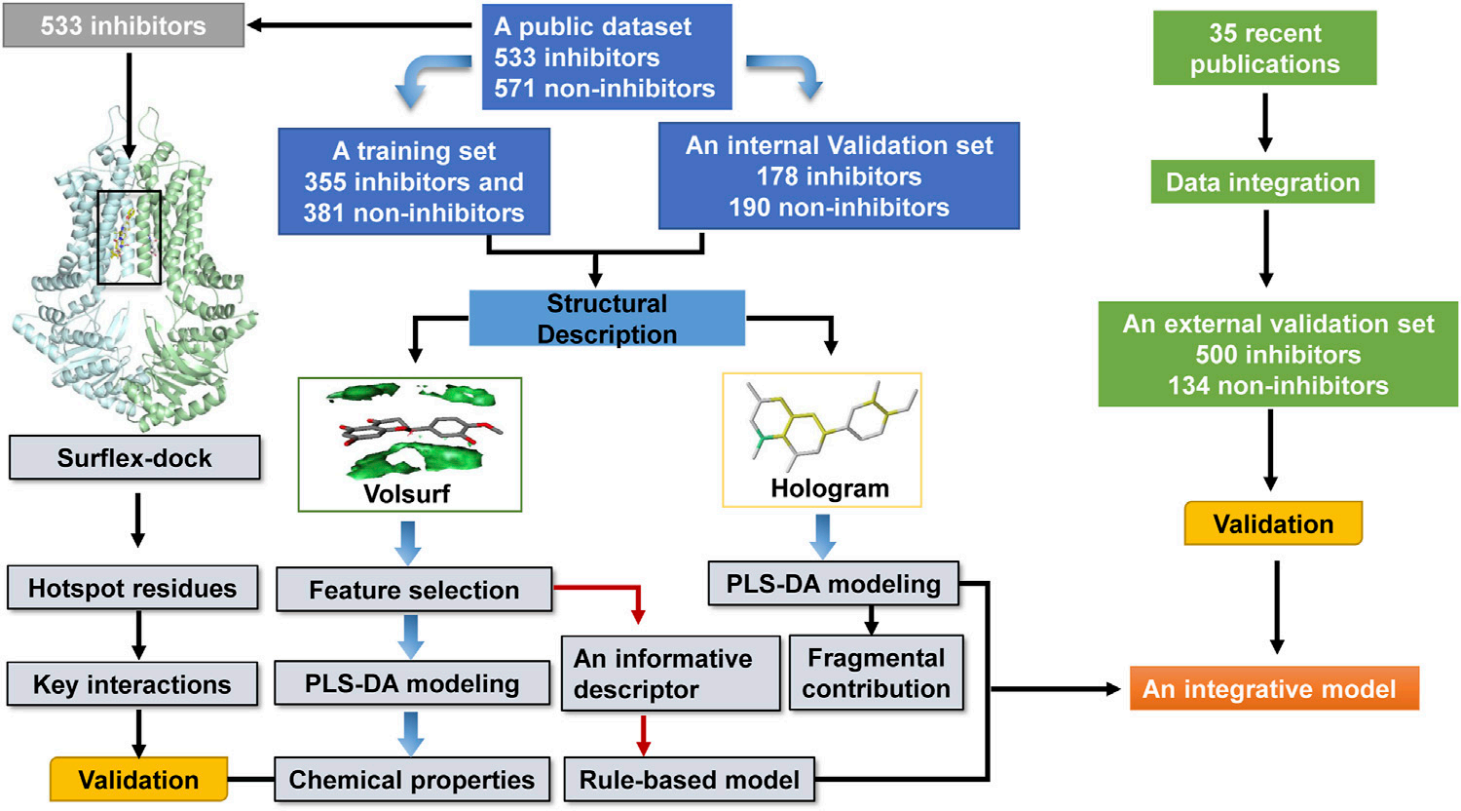
Schematic representation of the methods employed in our modeling.

A schematic overview of our modeling workflow, including the PLS-DA model and others, is given in Figure 1. Prior to training volsurf-based models, stepwise linear regression was performed on the 118 VolSurf descriptors for variable selection, yielding nine variable subsets based on the 736 training compounds. Details of variable selection and description is shown in Table 1. In this case, no variable was excluded in each iteration. Then, 10 PLS-DA models were constructed by using full descriptors and nine variable subsets, and their performances are shown in Table 2. All the volsurf-based PLS-DA models performed well in predicting ABCG2 inhibition, with a predictive accuracy of higher than 70% on the training and internal validation sets. Significant changes in the performances were not observed along with the decrease of descriptors. Additionally, balanced prediction accuracies for the inhibitors and non-inhibitors were observed in all models, with small differences between SPE and SEN. Given the accuracy, complexity, and interpretability, the model using a subset of only two descriptors was chosen as the best model with an overall accuracy of 0.73 on the training set. A similar performance on the internal validation set was achieved, indicative of a good predictive ability of the selected best model. To validate the robustness of the best PLS-DA model, 1000-times repeated modeling with randomly divided training/validation sets were performed. The means of accuracy were 0.75 ± 0.009 and 0.75 ± 0.019 for training and internal validation sets, respectively (Supplementary Figure S1). These results suggest that the volsurf-based PLS-DA model is robust and the selected descriptors significantly contribute to the classification model for the discrimination of inhibitors and non-inhibitors. Details of the informative molecular properties were discussed in below.

**TABLE 1:** Details of feature selection by stepwise linear regression on Volsurf descriptors.

Iterations	Entered in Sequence	Removed	*R* ^*2*^	Adjusted *R* ^*2*^	Description
1	BV12-DRY	—	0.217	0.216	The best hydrophobic volumes generated by the hydrophobic probe at the energy level of −1.0 kcal/mol
2	Log*P*	no	0.254	0.252	Log of the octanol/water partition coefficient, which is computed by mean of a linear equation derived by fitting VolSurf descriptor to experimental data on water/octanol partition coefficient
3	W3-O	no	0.270	0.267	Hydrophilic regions generated by the carbonyl oxygen atom at energy level of −1.0 kcal/mol
4	D1-DRY	no	0.280	0.276	Hydrophobic regions generated by the hydrophobic probe at energy level of −0.2 kcal/mol
5	R-OH2	no	0.293	0.289	Ratio Volume/Surface generated by the water probe
6	W4	no	0.300	0.294	The hydrophilic regions, represent the molecular envelope accessible generated by solvent water probe at energy level of −2.0 kcal/mol
7	Emin1-OH2	no	0.304	0.298	Local interaction energy minima between the H2O probe and the target molecule
8	D12-DRY	no	0.309	0.301	Hydrophobic local interaction energy minima distances generated by the hydrophobic probe
9	W7-O	no	0.287	0.282	Hydrophilic regions generated by the carbonyl oxygen atom at energy level of −5.0 kcal/mol

**TABLE 2:** Performance of VolSurf-based PLS-DA models.

Model	Number of Descriptors	Training Set				Internal Validation Set			
		ACC	SEN	SPE	MCC	ACC	SEN	SPE	MCC
VS1	118	0.76	0.75	0.77	0.52	0.77	0.78	0.76	0.53
VS2	9	0.76	0.72	0.78	0.51	0.79	0.76	0.81	0.57
VS3	8	0.76	0.75	0.77	0.52	0.79	0.78	0.80	0.57
VS4	7	0.75	0.76	0.74	0.50	0.79	0.79	0.78	0.57
VS5	6	0.75	0.74	0.75	0.50	0.79	0.79	0.78	0.57
VS6	5	0.75	0.74	0.76	0.50	0.78	0.78	0.78	0.56
VS7	4	0.75	0.74	0.75	0.49	0.77	0.76	0.77	0.53
VS8	3	0.74	0.72	0.76	0.47	0.78	0.76	0.78	0.55
**VS9** **^a^**	**2**	**0.73**	**0.73**	**0.74**	**0.46**	**0.76**	**0.74**	**0.77**	**0.51**
VS10	1	0.71	0.69	0.75	0.43	0.77	0.74	0.80	0.54

a The best VolSurf-based PLS-DA model based on two descriptors was highlighted in bold.

#### 3.1.2 Hologram-Based Models

The generation of molecular holograms is mainly determined by three parameters: fragment size, fragment distinction, and hologram length. Herein, various combinations of the fragment parameters and hologram length were used to train the PLS-DA models. The optimal parameter combination and the best model were determined in two steps. According to our experience, fragment size of four to seven is optimal in most cases, which covers the most important chemical groups but also decreases the number of fragments. Thus, the fragment distinction was first optimized with the fixed fragment size of 4–7. Table 3 shows the performance of 10 hologram-based PLS-DA models (FD1-FD10) using different kinds of fragments. Overall, the 10 models achieved satisfactory predictive performances. The statistical measures (ACC, SEN, and SPE) of five models were greater than 0.80 on the training and internal validation sets. Based on the prediction accuracies and model complexities, the model FD4 was selected as the best model in the first step, and that the atom and connection (A/C) was used to further optimize the PLS-DA models in the next.

**TABLE 3:** Performance of the PLS-DA models based on different fragment distinctions.

Model	Fragment distinction^a^	HL^b^	Training Set				Internal Validation Set			
			ACC	SEN	SPE	MCC	ACC	SEN	SPE	MCC
FD1	A	353	0.81	0.82	0.80	0.63	0.78	0.78	0.78	0.56
FD2	B	353	0.77	0.74	0.79	0.54	0.76	0.76	0.75	0.52
FD3	A/B	353	0.84	0.81	0.87	0.68	0.81	0.81	0.81	0.61
FD4	A/C	257	0.83	0.83	0.83	0.66	0.82	0.83	0.81	0.63
FD5	C/D	353	0.84	0.81	0.86	0.67	0.78	0.82	0.75	0.57
FD6	A/B/C	353	0.84	0.83	0.86	0.69	0.82	0.83	0.81	0.63
FD7	A/B/H	353	0.84	0.82	0.85	0.67	0.78	0.82	0.74	0.56
FD8	A/C/D	307	0.85	0.82	0.87	0.69	0.81	0.80	0.82	0.61
FD9	A/B/C/D	353	0.85	0.83	0.87	0.71	0.82	0.82	0.82	0.63
FD10	A/B/C/H	257	0.83	0.82	0.85	0.67	0.78	0.81	0.75	0.56

a The fragment distinction includes A (Atom), B (Bond), C (Connection), D (Donor & Acceptor), and H (Hydrogen Atoms).

b HL, holographic length.

By employing different fragment sizes combined with the A/C distinction, another 10 models (FS1-FS10) were constructed, with overall accuracies in the 0.79–0.86 range for the training set and 0.79–0.82 range for the internal validation set (Table 4). Among them, no significant difference in the performance metrics was observed, indicating that the fragment size had little effect on the model performances. By comparison, the performance of model FS5 based on the fragment size of five to eight was slightly better than other models. Accordingly, FS5 was selected as the best PLS-DA model, of which the ACC on the training and validation sets were 0.86 and 0.82, respectively. The best model performed very well in correctly identifying the inhibitors and non-inhibitors, with an identical prediction accuracy for both classes. Compared with the volsurf-based PLS-DA models, it is evident that hologram-based models displayed superior performance in discriminating ABCG2 inhibitors from non-inhibitors. When compared with self-reported model accuracy from previous studies, the validation set accuracy of our best hologram-based model is higher, to our knowledge, at most 78% previously.

**TABLE 4:** Performance of the PLS-DA models based on different fragment sizes.

Model	Atom counts	HL	Training Set				Internal Validation Set			
			ACC	SEN	SPE	MCC	ACC	SEN	SPE	MCC
FS1	1–4	151	0.79	0.78	0.81	0.59	0.79	0.79	0.78	0.57
FS2	2–5	257	0.81	0.80	0.82	0.62	0.81	0.85	0.78	0.63
FS3	3–6	353	0.83	0.83	0.83	0.66	0.80	0.82	0.78	0.60
FS4	4–7	257	0.83	0.83	0.83	0.66	0.82	0.83	0.81	0.63
FS**5** **^a^**	**5–8**	**353**	**0.86**	**0.85**	**0.86**	**0.71**	**0.82**	**0.82**	**0.82**	**0.63**
FS6	6–9	257	0.86	0.85	0.87	0.71	0.81	0.82	0.81	0.63
FS7	7–10	307	0.86	0.83	0.88	0.72	0.81	0.81	0.81	0.61
FS8	8–11	307	0.85	0.81	0.89	0.70	0.80	0.80	0.81	0.61

a The best Hologram-based PLS-DA model was highlighted in bold where the fragment size was 5–8, the fragment distinction was A/C, and the hologram length was 353 bins.

### 3.2 External Validation on Unknown Data

In real-world drug discovery, development of in silico models is usually aimed at identifying active compounds through virtual high-throughput screening from large chemical libraries. Therefore, the assessment of the actual behavior of a useable model against unknown data is indispensable. The most rigorous validation of model performance is to evaluate their robustness and predictive power on an external set, which is often lacking in the previous modeling studies. To address this issue in this wok, an in-house dataset containing 634 compounds was curated from recent *in vitro* experiments, which was used to further validate the effectiveness of our models. Somewhat surprisingly, both of the best volsurf- and hologram-based models achieved satisfying performances on the external set, with the prediction accuracies of 0.70 and 0.75, respectively (Table 5), confirming the capability of our models to generalize to unseen data. Nevertheless, the hologram-based PLS-DA model performed better in the external validation, implying that it may be more suitable for the virtual screening campaigns in drug discovery.

**TABLE 5:** Performance of the best models on the external validation set.

Best Models	TP	TN	FP	FN	ACC	SEN	SPE	MCC
VolSurf-based	327	116	18	173	0.70	0.65	0.87	0.43
Hologram-based	359	118	16	141	0.75	0.72	0.88	0.50

### 3.3 Chemical Properties Associated Molecular Interactions

**FIGURE 2:**
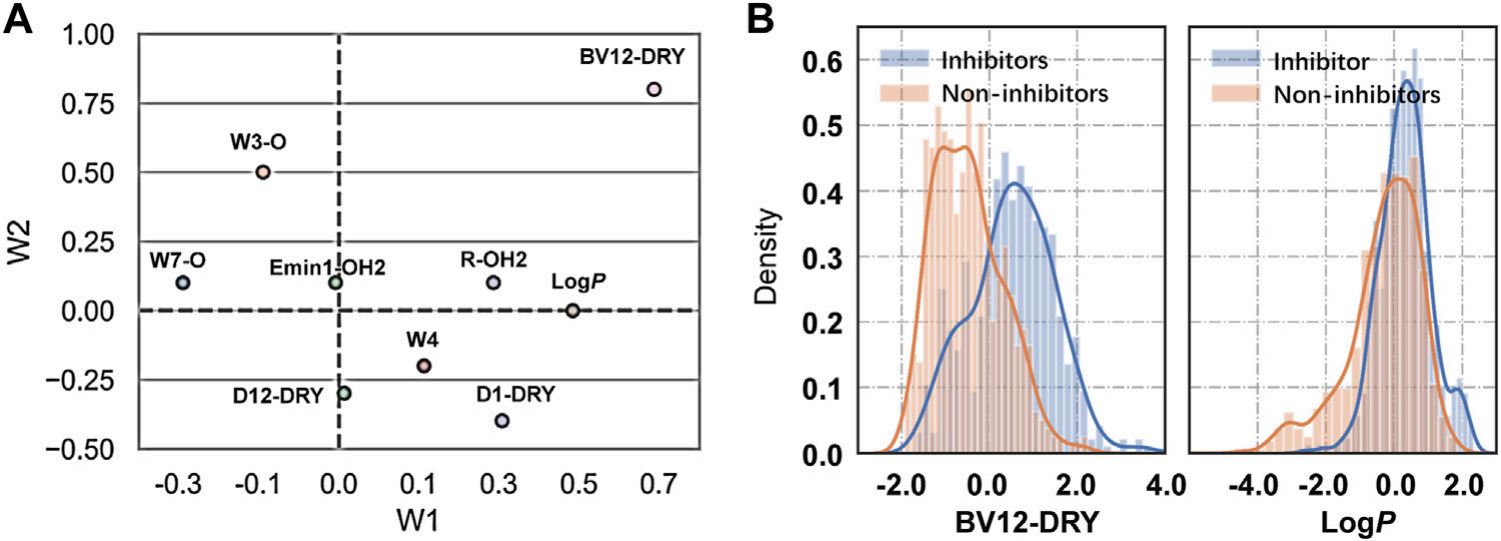
Analysis of VolSurf descriptors. **(A)** Loadings of the nine independent variables in the first two principal components. **(B)** Density distribution of the descriptors BV12-DRY (*p* = 1.566 × 10^–69^) and Log*P* (*p* = 8.054 × 10^–29^) in the two classes. The *p* values were calculated by using Student’s t-test.

The binding of drugs from the extracellular aqueous phase to the transporter cavity is a dynamic process including membrane partitioning and transporter binding steps, each of which was governed by distinctive molecular interactions. As described earlier, nine significant VolSurf descriptors were selected in sequence using stepwise linear regression analysis. The descriptor importance was examined by the correlation coefficients (*R* ^2^ or adjusted *R* ^2^) of the independent variables with the binary classes. It can be observed that there are no significant improvements on the correlations along with the sequential introduction of variables into the regression models (Table 1). From the loading plot of the nine independent variables in the first two principal components (Figure 2A), it can be also observed that BV12-DRY and LogP make leading contributions to the trained model VS2 (Table 2), consistent with the outcome of feature selection. The selected best volsurf-based model with good performance only employed the top two descriptors, thus being simple and strongly interpretable. Distributions of the two descriptors between inhibitors and non-inhibitors are significantly different (Figure 2B), suggesting that they are relevant for the discrimination of the two classes.

The water-octanol partition coefficient LogP (*p* = 8.054 × 10^–29^, *t* test), as a commonly used measure of molecular hydrophobicity, is a relevant molecular property for transporter inhibition, which has been claimed in previous studies (Matsson et al., 2007; Matsson et al., 2009; Nicolle et al., 2009; Sjöstedt et al., 2017). It is to be expected that the inhibitors are more hydrophobic, given the fact that a lipid-water partitioning step driven mainly by hydrophobic interactions is required before reaching the transporter binding site (Xu et al., 2015). However, the distribution of Log*P* between the two classes overlaps largely, thus not being able to distinguish inhibitors from non-inhibitors effectively. This suggested that the LogP was not highly informative, which in fact was declared as relevant when combined with other molecular features.

Compared with the LogP, the BV12-DRY is significantly more predictive (*p* = 1.566 × 10^–69^, *t* test). This descriptor represents the best hydrophobic volumes generated by the hydrophobic probe (DRY) at -1.0 kcal/mol. To the best of our knowledge, the predictive value of the BV12-DRY for ABCG2 inhibitors has not been discussed previously. Note that the “best volume” here is not in fact a measure of molecular size. Given the nature of VolSurf descriptors, it could be understood as the volume or the surface of the interaction contours presented in the 3D grid map. In the case of BV12-DRY, the interaction energy derived by the DRY probe involves not only hydrophobic interactions, but also electrostatic effects and hydrogen bonds. Thus, this descriptor can be regarded as an ensemble of the lipophilicity, the hydrogen bond acceptors and donors, and other electrostatic interactions. According to the distribution difference of BV12-DRY in the two classes (Figure 2B), an extremely simple rule derived from this descriptor can discriminate the majority of inhibitors and non-inhibitors correctly: compounds with BV12-DRY > −0.1 are very likely to be inhibitors whereas compounds with BV12-DRY < −0.1 are prone to be non-inhibitors. The rule-based model achieved an overall accuracy greater than 0.70 for the training and two validation sets (Table 6), which is comparable to the volsurf-based PLS-DA models. It means that the BV12-DRY, as a single feature is highly informative and predictive, which plays a dominant role in identifying ABCG2 inhibitors. In the two-step binding process, drug binding to the transporter was driven essentially by weak electrostatic interactions and hydrogen bonds (Matsson et al., 2007; Xu et al., 2015). Thus, the successful binding of an inhibitor to ABCG2 is determined not only by a proper membrane solubility but also by concordant electrostatic properties, further strengthening the key role of BV12-DRY in predicting transporter inhibition.

**TABLE 6:** Performance of the rule-based model on different data sets.

Data Sets	TP	TN	FP	FN	ACC	SEN	SPE	MCC
training	262	264	117	93	0.71	0.74	0.69	0.43
internal validation set	144	144	46	34	0.78	0.81	0.76	0.56
external validation set	338	111	23	162	0.71	0.68	0.83	0.42

### 3.4 Fragmental Contribution to Molecular Interactions

**FIGURE 3:**
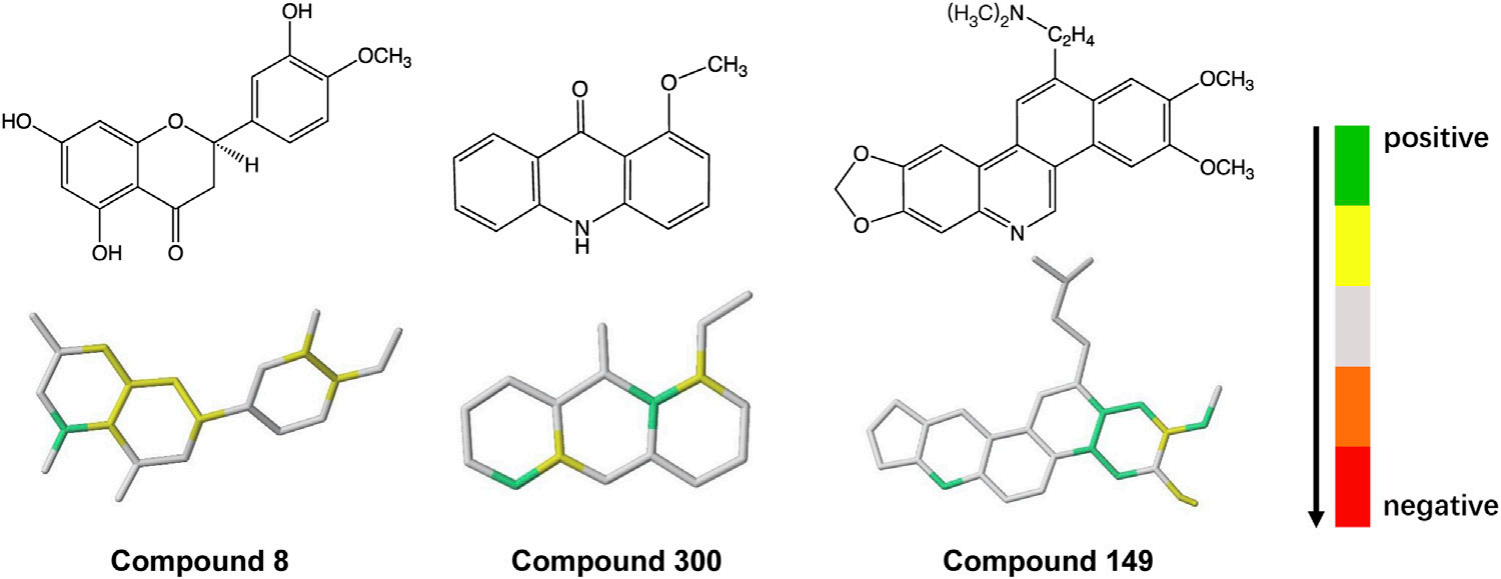
Mapping of atomic contributions in the fragments of ABCG2 inhibitors with diverse scaffolds.

To explore the molecular fragments contributing to ABCG2 inhibition, each fragment in the hologram was generated in turn, and the contribution to activity of each atom in the fragment was taken as the PLS coefficient divided by the number of atoms. The molecule was then color coded according to the individual atomic contributions. As shown in Figure 3, atoms in the aromatic biphenyl and benzoheterocycle (such as quinoline and chromene rings) have relatively high contribution, indicating that these fragments are favorable to ABCG2 inhibition. The abundant π-electron systems in the scaffolds may be responsible for the binding of inhibitors via *π*-*π* stacking interactions with the aromatic residues in the drug-binding pocket. The N and O atoms in the heterocycle or functional groups may form hydrogen bond interactions with the polar residues. These structural properties could provide medicinal chemists inspiration for the fragment-based design of novel lead compounds that might lead to suitable drug candidates.

### 3.5 Binding Mode Analysis by Molecular Docking

To validate the chemical and structural properties derived from the ligand-based models, structure-based molecular docking was performed to investigate the binding mode of ABCG2 inhibitors. In this work, Surflex-Dock method was used to simulate the binding of 533 inhibitors to the central cavity within the transmembrane domains. Before molecular docking, the co-crystallized inhibitor MZ-29 was firstly re-docked into the binding site of ABCG2. The top-scored docking pose of MZ-29 (score = 9.44) was superimposed with the crystal conformation very well with a RMSD (root-mean-square deviation) of 0.83 Å (Supplementary Figure S2), which suggested that the protocol of Surflex-dock can reproduce the native binding mode effectively.

**FIGURE 4:**
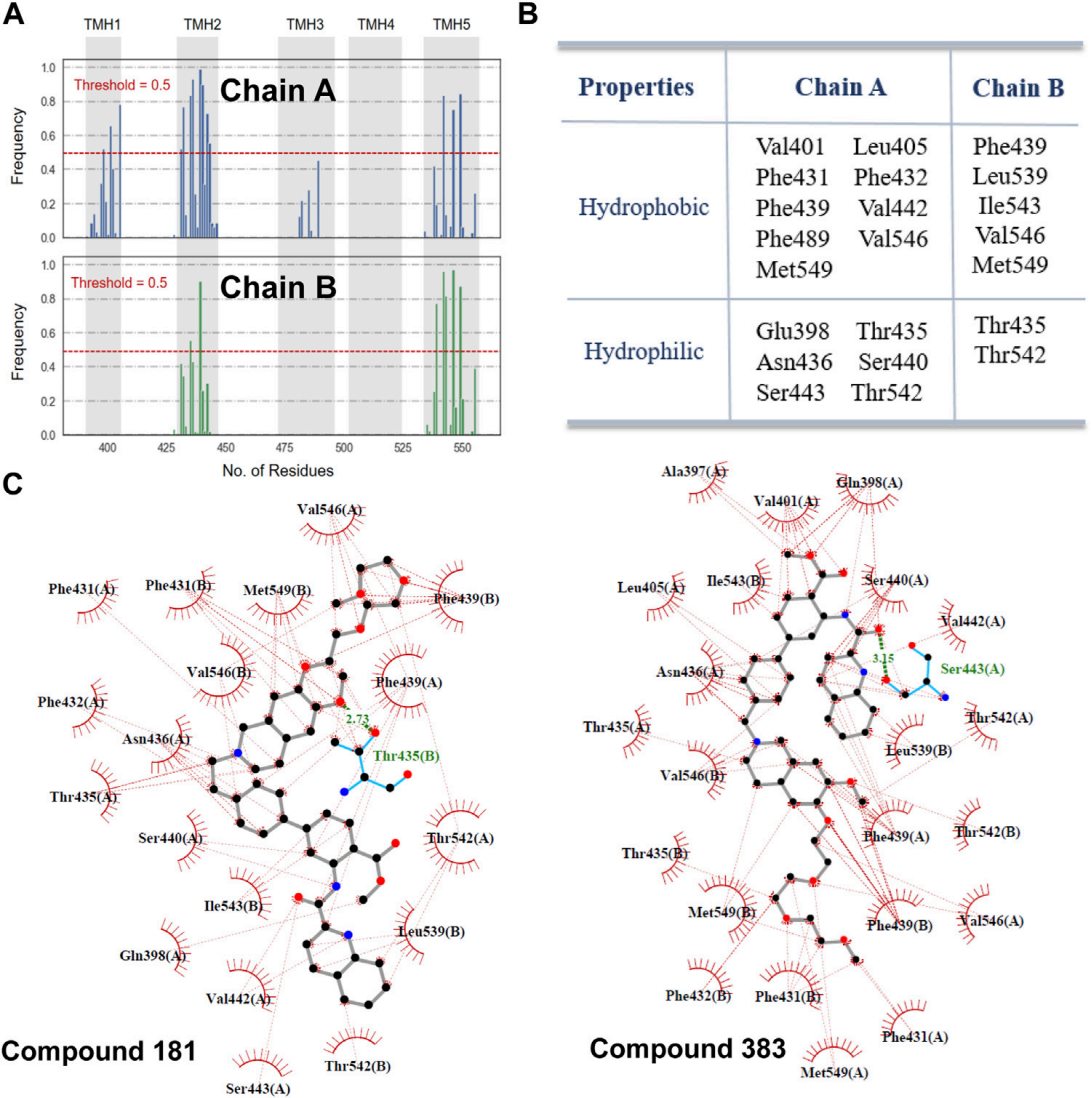
Molecular interactions with ABCG2. **(A)** The occurrence frequencies of the binding residues involved in the molecular interactions with the docked inhibitors. **(B)** 22 hotspot residues with a high-frequency occurrence (>0.5) intimately related to the binding of inhibitors. **(C)** Interactions of compound 181 and 383 with the residues in the binding pocket. H-bonds interactions are represented as green dashed lines.

Figure 4 lists the hotspot residues with high occurrence frequencies (>0.5) involved in the interactions with the docked inhibitors. The inhibitor-binding pocket is mainly constituted by a large number of lipophilic residues and a pool of hydrophilic residues (Figures 4A,B). Herein, two compounds with strong inhibitory activities at nano-molar level were selected for exploring the interactions with the highly related residues. As shown in Figure 4C, the two inhibitors embed well in the binding pocket by forming strong hydrophobic interactions with the aliphatic and aromatic residue, including Val401, Leu405, Val546, Phe432 and Phe439. Additionally, it can be observed that *π*-*π* interactions with Phe439 and H-bond interactions with Thr435 and Ser443 were formed, respectively, which may contribute to the high binding affinity between inhibitors and the transporter. As proposed by Xu et al. (Xu et al., 2015), these interactions may play a critical role in transporter binding, further confirming the importance of the chemical and structural properties derived for ABCG2 inhibition. However, it is important to note that although the docking procedure could provide a glimpse of the binding profiles of ABCG2 inhibitors, the precise molecular mechanisms underlying the complicated binding process and specific ligand interactions need to be further probed by other computational techniques such as molecular dynamics simulations and enhanced sampling algorithms, which is beyond the scope of this work.

### 3.6 Model Improvement by an Integrative Approach

**FIGURE 5:**
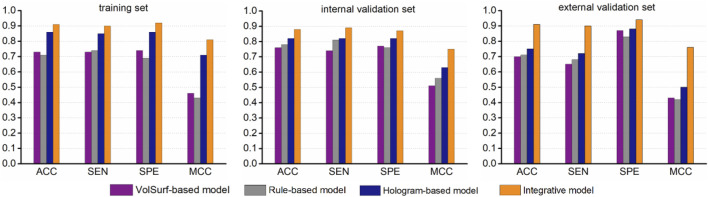
Comparison of the performance of the integrative model with the single classifiers.

While the best hologram-based model performed well in predicting ABCG2 inhibitors, there was still a number of compounds were misclassified, particularly for the two validation sets. One possible explanation is that some specific structural fragments and molecular properties that is favorable to the interactions with the transporter can not be fully characterized by molecular holograms. Therefore, we hypothesized that specific physicochemical properties might be complementary to the fragmental descriptors, which could further fine-tune the model performance. To test this hypothesis, the misclassified compounds (false positives and false negatives) were revaluated based on the simple rule derived from the most informative VolSurf descriptor BV12-DRY. Surprisingly, according to the rule (BV12-DRY > −0.1 for inhibitors and < −0.1 for non-inhibitors), a large portion of misclassified compounds can be correctly predicted as true positives and true negatives, thus resulting in a significant improvement of the statistical support by at least 5% for model prediction on all data sets (Figure 5). We therefore proposed an integrative modeling approach for differentiating the inhibitors from non-inhibitors of ABCG2, in which the first step was conducted by PLS-DA combined with the fragment-based molecular hologram, followed by a fine-tuned step using the simple rule derived from the informative VolSurf descriptor. Collectively, the integrative model with the highest accuracy could be applied in the virtual screening and molecular design of potent compounds to modulate the efflux of the transporter.

## 4 Conclusion

The inhibition of ABCG2 can affect the ADMET characteristics of drugs, which was also considered as a promising strategy to enhance the efficiency of chemotherapy in cancer treatment. Thus, prediction of ABCG2 inhibitors is of paramount importance in drug development. Our work provided a number of simple and accurate models for predicting ABCG2 inhibition, as well as the key chemical and structural properties underlying specific molecular interactions. First, many in silico models were established by PLS-DA modeling technology in combination with VolSulf descriptors or molecular hologram based on a publicly available dataset. The best volsurf- and hologram-based models with good performance are simple, robust, and interpretable. Then, a straightforward rule-based model was derived from the most informative VolSurf property. The developed models performed well on an external set that was curated from the recent *in vitro* studies, further confirm the effectiveness of our models. Finally, we proposed an integrative model with superior performance for predicting ABCG2 inhibitors by combining a hologram-based PLS-DA model with the simple descriptor-based rule, which could provide a powerful cheminformatics tool for the evaluation of drug-ABCG2 interactions and molecular design of novel inhibitors.

## Data Availability

The original contributions presented in the study are included in the article/Supplementary Material, further inquiries can be directed to the corresponding authors.

## Supplementary Material

Supplementary Figure S1 The accuracy distributions of 1000-times repeated PLS-DA modeling with randomly divided training/validation sets.

Supplementary Figure S2 Evaluation of the Surflex-dock protocol. **(A)** Alignment of the top-scored docking pose (yellow) of the inhibitor MZ-29 (score = 9.44) on the native conformation (grey) with a RMSD of 0.83 ;Å. **(B)** Crucial residues involved in the molecular interactions with ABCG2 are shown in sticks. The hydrogen bonds are shown in black dashed lines.

Supplementary Table S1 Data sets of ABCG2 inhibitors and non-inhibitors used for the training and validation of in silico models.
