# Supplementary figures and images for: Development of Simple and Accurate in Silico Ligand-Based Models for Predicting ABCG2 Inhibition
Source: Front Chem. 2022 May 18;10:863146. doi: 10.3389/fchem.2022.863146 (PMC9159808; doi:10.3389/fchem.2022.863146)

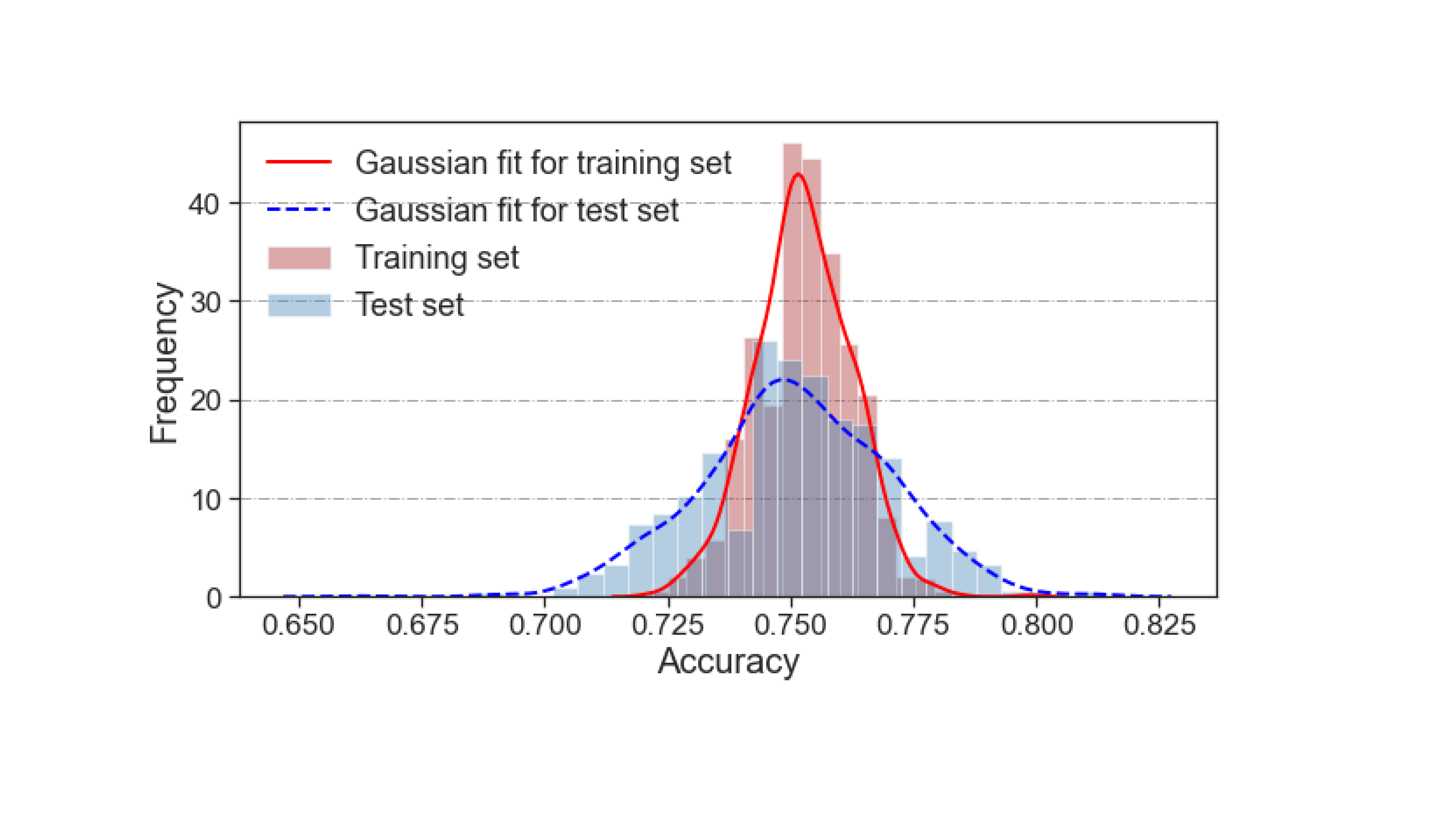

Supplement: Supplementary file 1 [file Image1.TIFF]

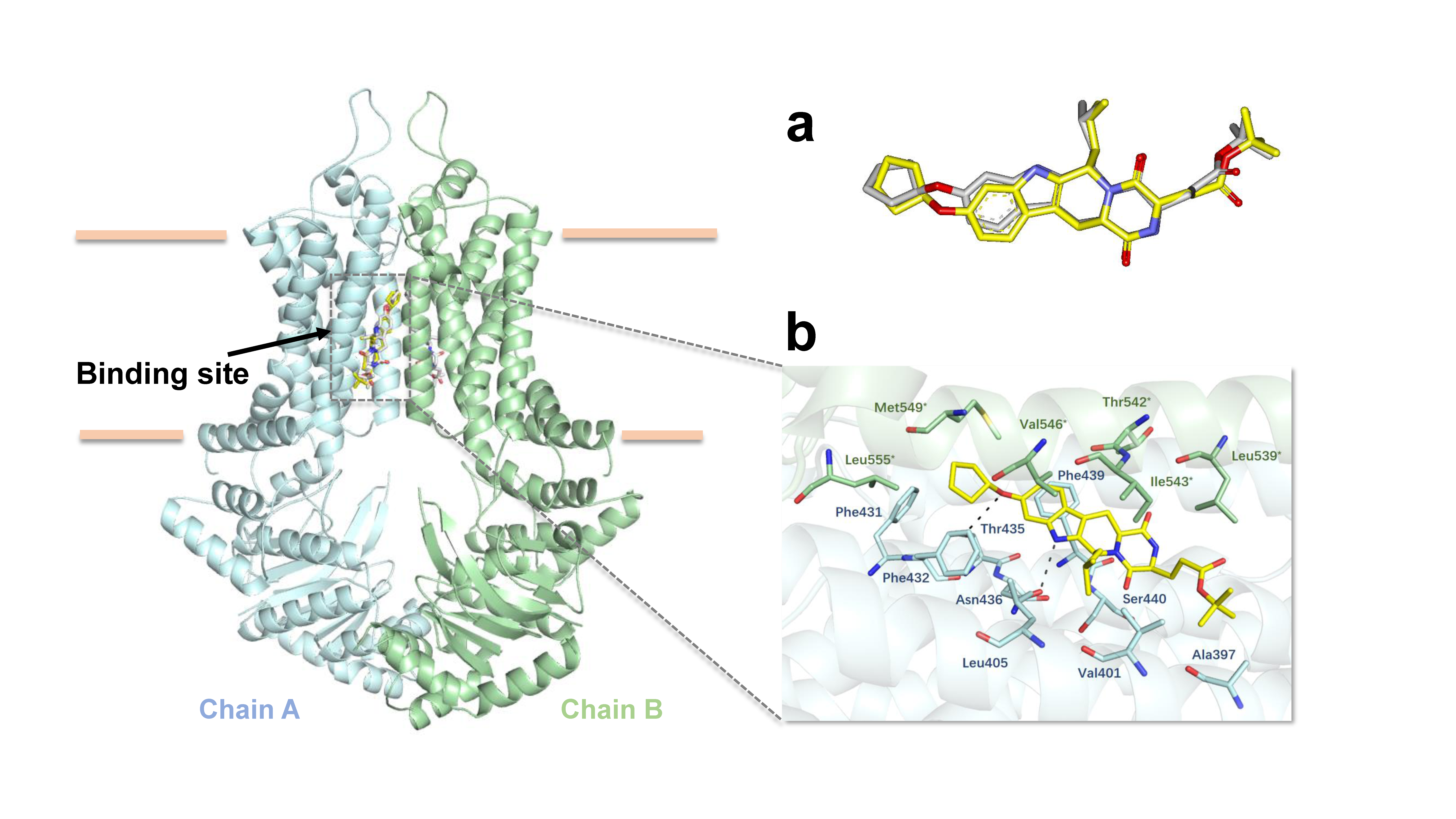

Supplement: Supplementary file 2 [file Image2.TIFF]
